# Influence of Methylene Blue on Microglia-Induced Inflammation and Motor Neuron Degeneration in the SOD1^G93A^ Model for ALS

**DOI:** 10.1371/journal.pone.0043963

**Published:** 2012-08-27

**Authors:** Payam Dibaj, Jana Zschüntzsch, Heinz Steffens, Jörg Scheffel, Bettina Göricke, Jochen H. Weishaupt, Karim Le Meur, Frank Kirchhoff, Uwe-Karsten Hanisch, Eike D. Schomburg, Clemens Neusch

**Affiliations:** 1 Max-Planck-Institute for Experimental Medicine, Göttingen, Germany; 2 Department of Neurology, University of Göttingen, Göttingen, Germany; 3 Institute of Physiology, University of Göttingen, Göttingen, Germany; 4 Institute of Neuropathology, University of Göttingen, Göttingen, Germany; 5 Department of NanoBiophotonics, Max-Planck-Institute for Biophysical Chemistry, Göttingen, Germany; 6 Department of Neurology, University of Ulm, Ulm, Germany; 7 Department of Molecular Physiology, University of Saarland, Homburg, Germany; 8 Systems Neuroscience Group, J. F. Blumenbach Institute of Zoology and Anthropology, University of Göttingen, Göttingen, Germany; Virginia Commonwealth University, United States of America

## Abstract

Mutations in SOD1 cause hereditary variants of the fatal motor neuron disease amyotrophic lateral sclerosis (ALS). Pathophysiology of the disease is non-cell-autonomous, with toxicity deriving also from glia. In particular, microglia contribute to disease progression. Methylene blue (MB) inhibits the effect of nitric oxide, which mediates microglial responses to injury. *In vivo* 2P-LSM imaging was performed in ALS-linked transgenic SOD1^G93A^ mice to investigate the effect of MB on microglia-mediated inflammation in the spinal cord. Local superfusion of the lateral spinal cord with MB inhibited the microglial reaction directed at a laser-induced axon transection in control and SOD1^G93A^ mice. *In vitro*, MB at high concentrations inhibited cytokine and chemokine release from microglia of control and advanced clinical SOD1^G93A^ mice. Systemic MB-treatment of SOD1^G93A^ mice at early preclinical stages significantly delayed disease onset and motor dysfunction. However, an increase of MB dose had no additional effect on disease progression; this was unexpected in view of the local anti-inflammatory effects. Furthermore, *in vivo* imaging of systemically MB-treated mice also showed no alterations of microglia activity in response to local lesions. Thus although systemic MB treatment had no effect on microgliosis, instead, its use revealed an important influence on motor neuron survival as indicated by an increased number of lumbar anterior horn neurons present at the time of disease onset. Thus, potentially beneficial effects of locally applied MB on inflammatory events contributing to disease progression could not be reproduced in SOD1^G93A^ mice via systemic administration, whereas systemic MB application delayed disease onset via neuroprotection.

## Introduction

Amyotrophic lateral sclerosis (ALS) is an adult-onset neurological disorder characterized by progressive loss of upper and lower motor neurons. Transgenic mice and rats expressing various human ALS-linked mutations in the gene encoding the enzyme superoxide dismutase-1 (mSOD1) recapitulate to some extent the fatal paralysis seen in patients [1], [2], [3], [4], [5], [6]. Dominant mutations in SOD1 are a frequent cause of familial ALS (fALS). Additionally, the best-studied animal model of fALS is that caused by mutations in SOD1 [7]. In this context, recent clinical and electrophysiological data show that the human SOD1-G93A phenotype closely resembles sporadic ALS (sALS) implicating comparable disease pathology [8]. The mSOD1-mediated toxicity is non-cell-autonomous deriving not only from motor neurons but also from neighboring glia. In particular, microglia and astrocytes substantially contribute to motor neuron death and disease progression [1], [9], [10], [11], [12]. In the case of microglia, selective silencing of the mutant gene in these immune cells or replacement of mSOD1-expressing cells of the myeloid lineage including microglia by non-mutated cells via bone marrow transplantation substantially slowed progression of the disease [1], [5], [9]. Such studies strongly emphasize a microglial contribution to the progression of motor neuron death and disease progression. Despite this clear evidence for a neuroinflammatory role in neurodegeneration, anti-inflammatory therapies in mice and rats as well as in human trials have been disappointing.

Local tissue damage within the CNS immediately attracts microglial processes and the subsequent migration of ameboid transformed microglia and phagocytosis [13], [14], [15], [16], [17]. Nitric oxide (NO), beside other signal molecules, plays a crucial role as a chemoattractant and activator of microglia [16], [18]. Spinal superfusion with inhibitors of NO synthase (NOS) and soluble guanylate cyclase (sGC), the main target enzyme of NO, e. g., with the dye methylene blue (MB), efficiently inhibits injury-directed reactions of microglia at least in the superficial layers of the dorsal column as a sensory division of the spinal cord [16]. In addition, microglia in SOD1^G93A^ mice show substantial differences in inflammatory activity within the affected lateral column of spinal cord when compared to control mice [19], namely highly reactive microglia in pre-clinical stages and ameboid transformed and activated microglia with reduced injury-directed response in clinical stages.

MB, a phenothiazine compound, has been used to treat malaria, is the first line treatment of methemoglobinemia and is frequently used in the treatment of ifosfamide-induced encephalopathy [20]. Recently, beneficial effects in Alzheimer's disease (AD) have been reported [20], [21], [22]. Here, we used transgenic mouse combined with time-lapse 2-photon laser-scanning microscopy (2P-LSM) to investigate the effect of MB on microglial behavior in the SOD1-G93A (SOD1^G93A^) mouse model for ALS. MB-treated animals were subjected to *in vivo* imaging to study MB effects on microglial behavior at a cellular level in an intact environment within lateral tracts of the spinal cord, which largely convey efferent signals, i. e. for motor functions. To confirm local anti-inflammatory effects, we analyzed the influence of MB on cytokine and chemokine release from microglia of control and advanced clinical SOD1^G93A^ mice *in vitro*. Furthermore, we investigated the effect of MB on disease onset, progression and survival as well as on the motor behavior in the ALS model. Additionally, we performed immunohistochemical studies with regard to microglia and motor neurons.

## Materials and Methods

### Ethics statement

The experiments were performed according to the ethical guidelines of the national animal protection law and were authorized by the ethical committee of the State of Lower Saxony (review board institution: Niedersächsisches Landesamt für Verbraucherschutz und Lebensmittelsicherheit, Dezernat 33, Oldenburg, Germany; approval-ID: 509.42502/01-39.03). Furthermore, the ARRIVE Guidelines (Animal Research: Reporting *In Vivo* Experiments; NC3Rs) have been followed.

### Mouse strains and motor tests

Mice transgenic for the mutated human SOD1^G93A^ (TgN[SOD1-G93A]G1H) were originally obtained from the Jackson Laboratory, USA (strain: B6.Cg-Tg(SOD1-G93A)1Gur/J) and bred at the Max Planck Institute (MPI) for Experimental Medicine Göttingen. Transgenic mice were maintained in the hemizygous state by mating G93A males with B6SJL hybrid females for more than 10 generations. Animals were fed standard AIN93G diets. Drug administration was orally in drinking water or by intraperitoneal (i. p.) injection, started at the age of 8 (oral) or 7 (i. p.) weeks. Neuropathology is first detected at this time in SOD1^G93A^ mice but without clinical expression of disease [23], [24]. The oral study used 10 mice per group that were treated with MB (Sigma-Aldrich, Munich, Germany) 3, 10, 30 and 100 mg per kg body weight per day. The control group consisted of 16 mice on a standard diet. The distribution of sexes was similar in each group. Water consumption was monitored in 1–2 mice from each group throughout the oral experiment in the different clinical stages. The i. p. study used 16 mice per group and received either a daily injection of 0.9% NaCl solution alone as control or 10 mg MB per kg per day dissolved in 0.9% NaCl. The distribution between the sexes was also similar in both groups.

**Figure 1:**
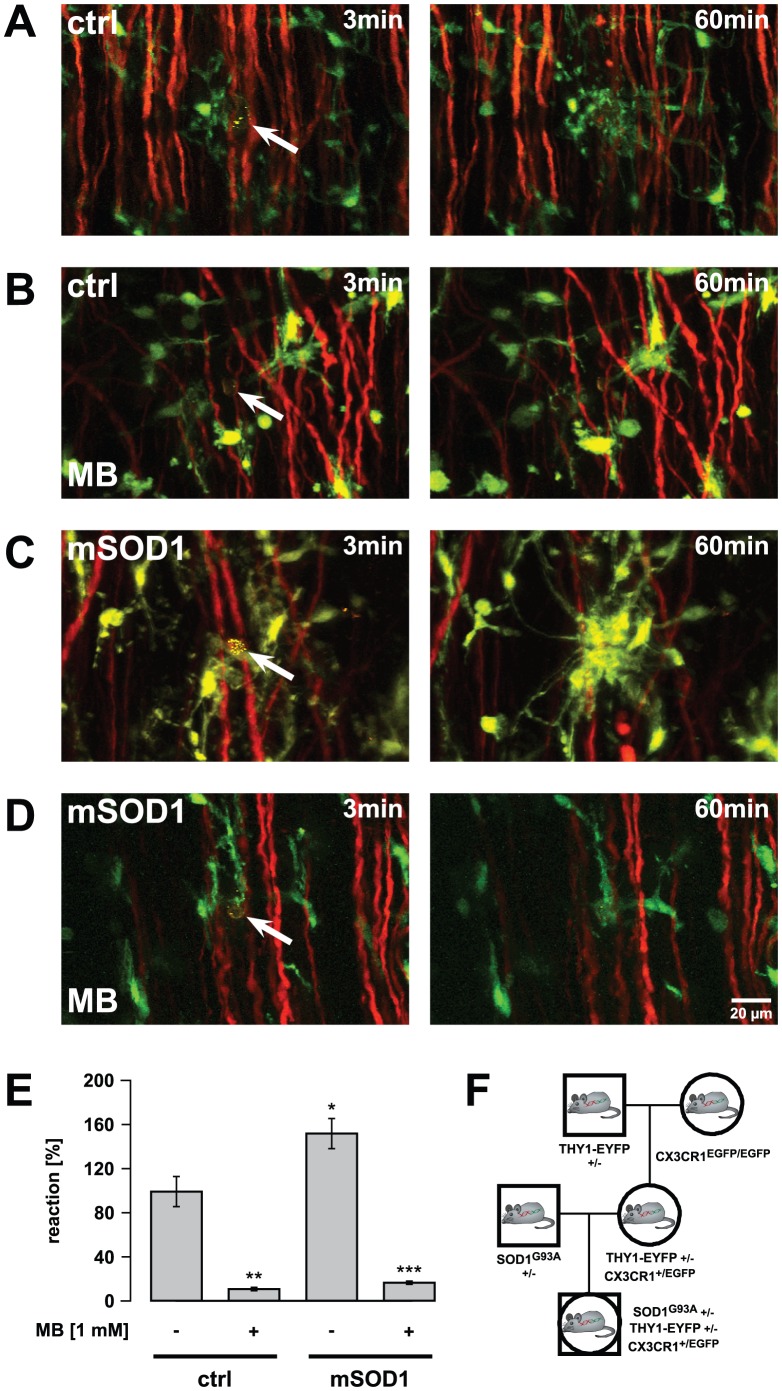
Inhibition of microglial reaction towards axonal injury in the lateral column by local application of MB *in vivo.* Microglial reaction towards laser-induced axonal transections within the lateral column of the spinal cord was recorded. Tissue injuries were induced by high-power laser pulses. The experiments have been performed in double transgenic mice expressing EGFP in microglia and EYFP in projection neurons. For better visualization, EYFP fluorescence in the images is depicted with a red colour table. Images are arranged such that rostral is to the upper side. (A-D) Left images were taken immediately (3 min) after axonal transection (autofluorescence and arrow) in control and mutant (SOD1^G93A^) mice. Respective images (right) were taken 60 min after injury. In respective experiments spinal cord was superfused with MB (1 mM). (E) Quantification of microglial response (increase of EGFP fluorescence around the injury) to the injured site. Control and mutant mice were of corresponding age (60 to 90 days of age). n = 7 mice for control response, n = 4 for MB-modified response in control mice, n = 9 for response in SOD1^G93A^ mice, n = 7 for MB-modified response in SOD1^G93A^ mice. (F) Breeding strategy to obtain SOD1^G93A^ mice with fluorescently labeled microglia and projection neurons. Values are presented as mean ± SEM; ANOVA followed by Tukey test (*p<0.05, **p<0.01, ***p<0.001).

For 2P-LSM experiments, hemizygote male TgN(SOD1-G93A) mice (B6SJL background for more than 10 generations) were crossbred with female TgH(CX3CR1-EGFP)xTgN(THY1-EYFP) mice to obtain SOD1^G93A^ mice with fluorescently labeled microglia and projection neurons (Fig. 1F), [19], [25]. Double-transgenic TgH(CX3CR1-EGFP)xTgN(THY1-EYFP) mice were previously obtained by crossbreeding homozygous CX3CR1-EGFP mice, in which the expression of EGFP in microglia is achieved by inserting the EGFP reporter gene into the *Cx3cr1* locus encoding the chemokine receptor CX3CR1 [26], with transgenic THY1-EYFP mice expressing the yellow fluorescent protein EYFP in projection neurons and their respective axons (Fig. 1F), [27]. TgH(CX3CR1-EGFP) mice and TgN(THY1-EYFP) mice were of B6SJL background for more than 10 generations. The corresponding littermates (hemizygotes with respect to all three genetic alterations) were used to study microglial behavior *in vivo.* Even though female SOD1^G93A^ expressing mice survive few days longer than male mutant mice, gender-dependent differences with regard to the effect of MB on mSOD1 disease progression, and especially on microglial behavior were not observed. Accordingly, no gender differences in the progressive loss of functional motor units is observed in the SOD1^G93A^ mice [28]. The *in vivo* experiments were carried out on adult mutant mice and non-transgenic littermates (referring to the SOD1 gene; control). In control mice no age-related changes in microglial behavior were observed within the studied ages (adult animals). In treated mice, which were used for *in vivo* imaging and immunohistochemical investigations, drug administration started at the age of 7 weeks.

SOD1^G93A^ mice monitored in this study showed first signs of disease at about 100 days of age and reached end stage disease at about 130 days of age. The mice were classified as ‘pre-clinical’ when showing no signs of motor deficits; early clinical stage (“stage 1”, onset of disease) when an abnormal gait with limb tremor was first apparent corresponding to an age of about 105 days; clinical stage (“stage 2”, about 115 days) when paralysis of at least one limb was observed; and advanced clinical stage (“stage 3”, about 123 days) when righting reflex failure occurred owing to the complete paralysis of hind limbs and at least one forelimb [29]. Animals were sacrificed when they were no longer able to right themselves within 10 s of being placed on their sides.

Motor performance was evaluated by means of a rotarod task. Mice were placed on a horizontal rod that was made to rotate either constantly at 12 rounds per minute (rpm) or at 1 rpm with an acceleration rate of 1 rpm every 10 s until the mouse fell off. Each mouse was tested three times per trial. Rotarod tests were started at the age of 60 days with subsequent tests at 7-day intervals. Grip strength of the forelimbs was also measured by a grip-strength-meter (TSE Systems, Bad Homburg, Germany) [30], [31], [32]. Mice held the grip and were pulled steadily backwards by the tail, the grip braking force being measured and expressed as milli-newtons per gram body weight. Each mouse was tested three times per trial.

### Immunohistochemistry

The animals were anesthetized (80 mg pentobarbital sodium per kg body weight i. p.) and perfused transcardially with PBS followed by 4% paraformaldehyde. After tissue processing, 20 µm-thick cryosections were cut from the lumbar spinal cord (level L3 to L5). After thawing and rehydration, the sections were incubated with blocking buffer (5% normal goat serum and 0.1% Triton X-100). The following primary antibodies were used: NeuN (rabbit, 1∶100; Millipore, Temecula, USA) and SMI-32 (mouse, 1∶1000; Sternbergs monoclonal, Emeryville, California, USA) to label neurons, Iba1 to label microglia (rabbit, 1∶500; Wako pure chemical Industries, Osaka, Japan), phospho-TDP-43 (pS403/404; rabbit, 1∶500; Cosmo Bio Co LTD, Tokyo, Japan) and SOD1 (rabbit, 1∶400; Enzo, Lörrach, Germany). Secondary goat anti-rabbit antibody labeled with Cy3 (1∶500; Millipore, Temecula, California, USA) was used for NeuN and Iba1. SOD1-immunoreactivity was developed with new fuchsin (Serva, Heidelberg, Germany) after 1 h incubation with AP (goat anti-rabbit, 1∶ 50; Dako, Hamburg, Germany) according to the manufacturer's instructions. For double labeling experiments, anti-SMI32 and anti-phospho-TDP-43 were used. Immunoreactivities were detected by appropriate Alexa 488 (Invitrogen, Camarillo, California, USA) and Cy3-conjugated secondary antibodies. Images were obtained using a Zeiss Axioplan 2 imaging fluorescence microscope (Zeiss, Jena, Germany) or a confocal laser-scanning microscope (Leica TSC SP2; Leica Microsystems Heidelberg GmbH) equipped with an acousto-optical beam-splitter and a 40x (NA 1.25) oil immersion objective. For detection of Alexa 488, a 488-nm Argon laser was used and emission was recorded between 505 and 521 nm. For detection of Cy3, excitation was performed by a 543-nm HeNe laser and emission was recorded between 553 and 630 nm. For quantification purposes of intracellular aggregates, analyze particle function of ImageJ (http://rsbweb.nih.gov/ij) was used.

To analyze motor neuron number, lumbar cross-sections of preclinical (90-day-old), clinical (110-day-old) and advanced clinical (about 130-day-old) mice were stained with NeuN and analyzed. Cell somata of NeuN-positive cells in the anterior horn which had a diameter larger than 20 µm were counted as these are probably motor neurons. In three lumbar sections approximately 100 µm apart both anterior horns were analyzed at 10x magnification for each mouse. The anterior horn was defined as the grey matter anterior to the middle of the central canal [33]. After counting the number of neurons within a grid of 0.4 mm×0.4 mm, these were expressed in terms of 1 mm^2^. These measurements probably included interneurons of relatively large size [34]. Tissue shrinkage and a possible loss of NeuN immunoreactivity during the disease course, as in the case of ischemia [35] or axotomy of the facial nerve [36], could have further distorted a true estimate number of motor neurons, especially in the advanced clinical stages. However, in the absence of specific motor neuron markers, we have accepted such inaccuracy in the comparison of cell numbers in the control and treated mice.

### Cytokine release assays

For the cytokine release studies on homogeneous cultures, control and SOD1^G93A^ microglia were plated in 96-well plates at a density of 1.5×10^4^ cells per well. As a control, microglia of newborn mice (ctrl_p0_) were used as previously described [37], [38]. Mutant microglia were obtained from whole spinal cord dissection of SOD1^G93A^ mice in advanced clinical stage (stage 3). For age-matched control, control microglia (ctrl_p120_) were obtained from corresponding adult siblings. After 1 day, cells were exposed either to fresh medium (basal release values) or medium containing LPS (lipopolysaccharide, final concentration 1 ng/ml) (*E. coli* R515 (TLR4) Axxora/Appotech TLR ligand set 1 (APO-54N-018)) or MB (1, 10, 20, 40 or 100 µM), or both. Cells were incubated for 18 h and supernatants analyzed. Cells were rinsed and new medium was added containing WST-1 agent for a viability assay (Roche Diagnostics). Measurements of cytokines and chemokines in culture supernatants were performed with either factor- and species-specific complete ELISA systems, or sets of capture and detecting antibody pairs, in addition to standard proteins (R&D Systems, Biolegend) as previously described in detail [37], [38]. Adaptations were made to reduce sample size and assay volume. Analyzed factors were TNFα, IL-6, IL-12p40, MCP-1, CXCL1 (KC, the mouse equivalent of GROα), CCL3 (MIP-1α) and CCL5 (RANTES).

### Anesthesia and surgery

*In vivo* experiments were carried out on respective transgenic SOD1^G93A^ and control mice of corresponding age under general anesthesia initiated by 80 mg/kg pentobarbital sodium i. p. (dissolved in 0.9% NaCl). After cannulation of the jugular vein, anesthesia was continued with 40–60 mg per kg and h methohexital sodium (Brevimytal, Hikma, London, UK). A tracheotomy was performed and a tube inserted for artificial ventilation. Dorsal and left lateral surface of spinal cord segments L4 and L5 were exposed by laminectomy of the spines L1 to L3 for imaging. The lateral column was imaged by rotating the mice by 80 degrees. Active respiratory movements were abolished by paralysis with pancuronium bromide (Pancuronium Organon, Essex Pharma GmbH, Munich, Germany; 800 µg per kg supplemented i. p. every hour) and artificial ventilation with a gas mixture of CO_2_ (2.5%), O_2_ (47.5%), and N_2_ (50%) at 120 strokes/min (100–160 µl/stroke depending on the body weight). The vertebral column was rigidly fixed with two custom-made clamps, each having two joints for keeping the mouse in the turned position. In all experiments, the exposed spinal cord was continuously superfused with ACSF (artificial cerebro-spinal fluid: 125 mM NaCl, 25 mM NaHCO_3_, 2.5 mM KCl, 1.25 KH_2_PO_4_, 1 mM MgCl_2_, 2 mM CaCl_2_*H_2_O and 10 mM glucose). For superfusion of the spinal cord with MB (solved in ACSF), the dura was carefully removed without lesioning the spinal cord. Rectal body temperature was measured and kept between 36 and 38°C by a heated plate. Electrocardiograms were monitored throughout the experiment. A heart rate maintained below 420 per min (also after noxious stimuli), as well as the need for high levels of heating were taken as signs for adequate anesthesia under paralysis and corresponded to the other reflex tests carried out before paralysis. Furthermore, care was taken that the dose of anesthesia during paralysis was equal to, or greater than, before paralysis [16], [17], [19], [25], [39].

### 2-Photon laser-scanning microscopy and image acquisition

High resolution *in vivo* imaging was performed using a commercial two-photon laser-scanning microscope (2P-LSM, Zeiss Axiocope 2 with LSM510 NLO scanhead) equipped with a fs-pulsed, mode-locked titanium-sapphire infrared laser (Mira 900/10 W Verdi; Coherent, Glasgow, UK) or a custom-made 2P-LSM equipped with a fs-pulsed titanium-sapphire laser (Chameleon Ultra II; Coherent). For 2P-recordings, a Zeiss W Plan Apochromat 20x (NA 1.0) water immersion objective was used. For excitation, the laser was set at 925±5 nm for the simultaneous excitation of EGFP and EYFP. Emitted light was split by a 520 nm longpass dichroic mirror (Semrock, Rochester, USA) and collected by photo-multiplier tubes (Hamamatsu, Japan) through two bandpass filters: a 494±20.5 nm (FF01-494/41-25) and a 542±25 nm (FF01-542/50–25), respectively (Semrock). The measured fluorescence intensities were comparable between the two microscopes. Parallel, uniformly spaced (1.5 to 2.4 µm) planes of 125*125 to 600*600 µm^2^ regions were recorded, digitized and processed to obtain z-stacks of images (256x256 to 1024x1024 pixels in size). Voxel sizes ranged from 0.24x0.24x1.5 to 1.17x1.17x2.4 µm for the xyz-axes. The total acquisition time for a stack of 15 to 30 images was approximately 1–2 min. Recordings of up to 100 µm stack depth were obtained. Stacks were acquired continuously to obtain time-lapse series of microglial action. Reproducible lesions were applied by the titanium-sapphire laser generally focused for about two seconds within an axon plane until fluorescence began to increase, resulting most often in transection of a single axon.

### Image processing and morphometric analysis

Image processing and morphometric analysis were performed using the Zeiss LSM software, ImageJ (http://rsbweb.nih.gov/ij) and Matlab (version 7, MathWorks, Ismaning, Germany). Statistical analysis was performed using Origin 7 software (Northampton, USA). Prior to any analysis, the time series of image stacks were corrected for the shifts in the horizontal and vertical directions by using an autocorrelation based custom-made software written in Matlab (v.7). For the majority of images background noise was removed by the median filter of ImageJ. The recorded stacks are shown in maximum intensity projections (MIPs). Although EGFP and EYFP signals cannot be separated without spectral unmixing, the filter sets were chosen in a way that permitted an unambiguous distinction of EGFP expressing microglia from EYFP expressing neurons. The cellular differentiation was facilitated by the different morphologies of axons and microglia and the predominance of EGFP signal in one channel. For quantification purposes we defined the microglial response as the increases in fluorescence directly around the injured site [14], [16], [19]. Whether such increases were a consequence of process ingrowth or soma immigration were not distinguished. We used the function *R*(*t*)  =  (*R*_x_(*t*)−*R*_x_(0))/*R*_y_(0) as described earlier [14], [16], [19]. To measure the microglial density around an injury we used the analyze particle function of ImageJ. Diameters for outer and inner area were 70 µm and 35 µm, respectively. Care was taken to include similar injuries in the different groups, indicated by the average size of the autofluorescence signal.

### Statistical analysis

For statistical analysis Origin 7 software (Northampton, USA) was used. Mean values are given ± standard of the mean (SEM). Statistical significance (p<0.05) was determined using ANOVA followed by the Tukey test.

## Results

### Inhibition of microglial activation by local application of MB *in vivo*

Inhibition of the NO pathway by local spinal application of different nitric oxide synthase (NOS) or soluble guanylate cyclase (sGC) inhibitors blocks microglial response to injuries within the superficial layers of the dorsal column as a sensory part of the spinal cord [16]. To test whether inhibition of the NO pathway influences injury-directed reactions of SOD1^G93A^ microglia within an affected motor part of the spinal cord, the exposed lateral column was superfused with MB, a broadband blocker of sGC and NOS [40]. As seen in Figure 1, superfusion with MB (1 mM) significantly suppressed the microglial response at the site of laser-induced axon transection within the lateral column to 12.2±1.9% of the control value set as 100±14.2% [19], a result comparable to the MB-induced suppression of microglial response in the dorsal column [16]. Figure 1A-B show a reduced accumulation rate of control microglia around the injured axon 60 min after a laser-induced lesion when the tissue was superfused with MB. Similar suppression was also observed in 60 to 90-day SOD1^G93A^ mice containing highly reactive microglia when the MB response of these microglia fell to 17.9±2.1% of the control value [19] (Fig. 1). Figure 1C-D also show a reduced rate of accumulation of modified microglia after injury when the tissue was exposed to MB. In the clinical stages, when microglia show reduced injury-induced reactions compared with control mice [19], these were also suppressed by locally applied MB (data not shown).

### Inhibition of cytokine/chemokine release from microglia by application of MB in vitro

**Figure 2:**
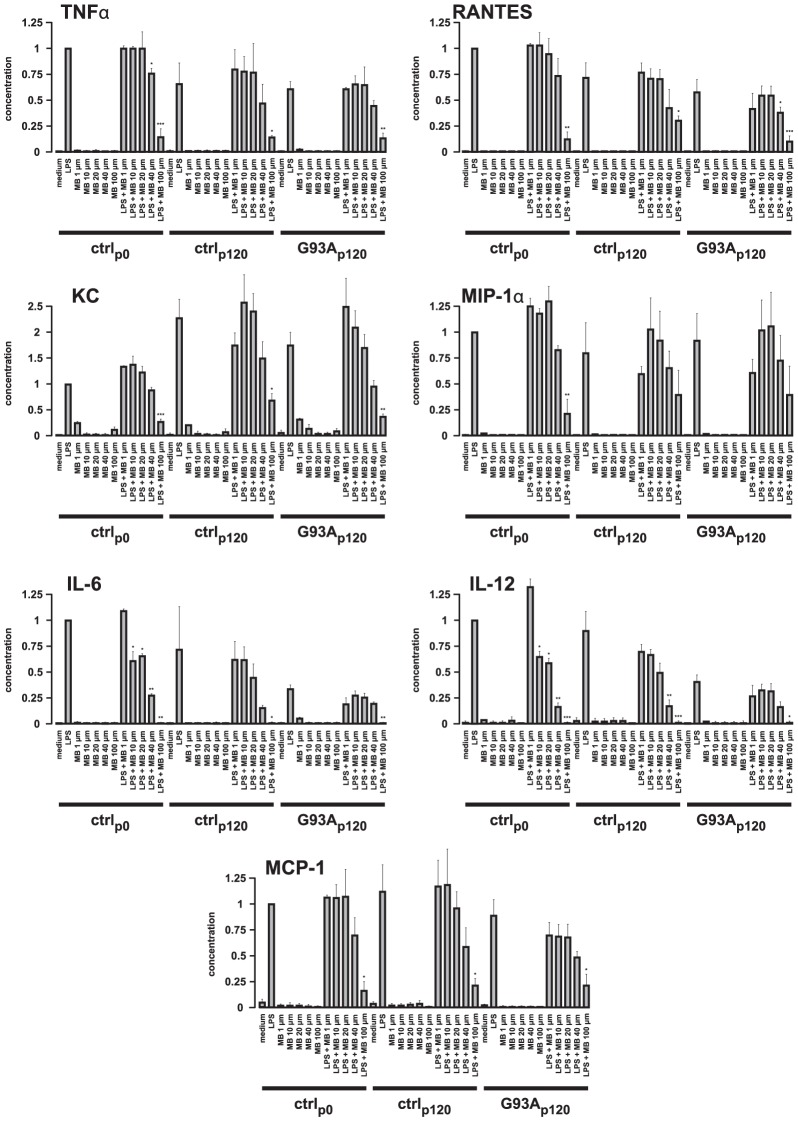
Inhibition of microglial cytokines and chemokines production by MB. Primary cultures of control (newborn control: ctrl_p0_; adult control from corresponding siblings of mutant mice: ctrl_p120_) or mutant (from advanced clinical SOD1^G93A^ mice; G93A_p120_) microglia (15,000 cells/well) were incubated with MB (1, 10, 20, 40 and 100 µM) in the presence or absence of LPS (100 ng/ml) for 18 hours. Cytokine and chemokine release profile was determined in the supernatants. Data are presented relative to the corresponding value of LPS-triggered cytokine or chemokine release from control microglia of newborn mice (set as 1). Data are mean ± SEM of triplicates of 3 (MCP-1), 4 (IL-6) or 5 (TNF-α, RANTES, KC, MIP-1α and IL-12) independent experiments; ANOVA followed by Tukey test (*p<0.05, **p<0.01, ***p<0.001).

To determine whether locally applied MB also suppresses the release of immunoregulatory mediators, thus to confirm an anti-inflammatory action of MB, we measured the secretion of different cytokines and chemokines by control (newborn mice: ctrl_p0_; age-matched controls (adult siblings): ctrl_p120_), and mutant microglia (advanced clinical mice: G93A_p120_). For this purpose, different concentrations of MB (1, 10, 20, 40 and 100 µM), alone or in combination with LPS, were added to microglia cultures and the cytokine/chemokine release was measured by ELISA. We measured the release of the following cytokines and chemokines: TNFα, RANTES, KC, MIP-1α, IL-6, IL-12 and MCP-1. The viability assay using WST-1 agent showed no significantly increased cellular death (data not shown). MB application alone induced no distinct release response, whereas stimulation by LPS resulted in a robust increase of all tested factors in control and mutant microglia (Fig. 2). LPS-induced release of all tested cytokines and chemokines from G93A_p120_ microglia was not distinctly different when compared with ctrl_p120_ microglia. We observed a clear decrease of TNFα, RANTES, IL-6 and IL-12 release from G93A_p120_ microglia when compared with the respective release from ctrl_p0_ microglia. This is probably due to altered expression levels in cultured microglia from adult mice or to a lower sensitivity to LPS of cultured microglia from adult mice. In contrast, the release of KC was increased from microglia of adult mice. The release of MIP-1α and MCP-1 was virtually unchanged.

Application of MB only at the highest concentration of 100 µM significantly reduced the LPS-triggered production of all tested cytokines and chemokines with the exception of MIP-1α from ctrl_p120_ or G93A_p120_ microglia, when compared with the LPS-triggered production from the same groups of microglia (Fig. 2). In the case of adult microglia, only the release of RANTES from G93A_p120_ microglia and of IL-12 from ctrl_p120_ microglia were significantly reduced by the application of 40 µM MB. Application of 1, 10 and 20 µM MB had no significant effects on cytokine and chemokine release. In the case of ctrl_p0_ microglia, the release of all tested cytokines and chemokines was significantly reduced by the application of 100 µM MB, the release of TNFα, IL-6 and IL-12 being already significantly reduced by the application of 40 µM MB and that of IL-6 and IL-12 by 10 and 20 µM MB (Fig. 2). Application of 1 µM MB had no significant effects on cytokine and chemokine release.

### Delayed onset of disease in MB-treated SOD1^G93A^ mice

**Figure 3:**
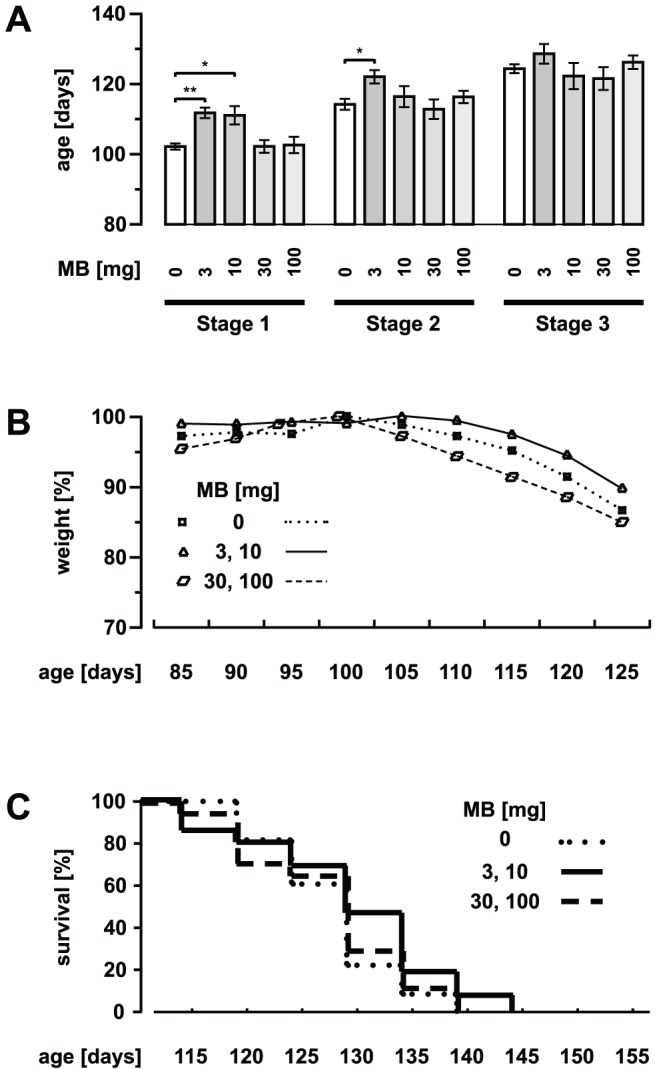
Delayed onset of disease by oral application of MB. (A) Control and MB-treated (3, 10, 30 and 100 mg oral per kg body weight per day) SOD1^G93A^ mice with respect to the different stages of disease (stage 1: onset of disease/early clinical stage; stage 2: clinical stage; stage 3: advanced clinical stage). (B and C) Weight and survival profiles for SOD1^G93A^ mice (control and MB-treated). In B the data are presented relative to the highest value in each group. Values are presented as mean ± SEM; ctrl: n = 16 mice, 3 mg: n = 12, 10 mg: n = 10, 30 mg: n = 8, 100 mg: n = 11; ANOVA followed by Tukey test (*p<0.05, **p<0.01).

MB has been used for a long time in various areas of biology and medicine, e. g. as first line treatment of methemoglobinemia [20] and recently beneficial effects of MB have been reported for clinical trials in AD [21], [22]. To test whether MB has a beneficial effect on mSOD1 disease course, they were orally treated with MB added to the drinking water (0, 3, 10, 30 and 100 mg per kg body weight per day). Given the knowledge about the well-studied and safe drug which has been also administered orally in human as well as about its high bioavailability [41], the oral route was chosen for MB administration. Whereas treatment with 3 or 10 mg MB significantly delayed the onset of disease compared to controls, treatment with 30 or 100 mg had no significant effect on disease onset (ctrl: 104.8±1.03 days; 3 mg: 112±1.64 days; 10 mg: 111.3±2.74 days; 30 mg: 102.5±1.95 days; 100 mg: 102.9±2.47 days) (Fig. 3A). Correspondingly, the onset of weight loss was delayed in mice treated with 3 or 10 mg MB (Fig. 3B), but weight loss was not itself significantly improved by MB. The progression to the later clinical stages (see Materials and Methods) was only delayed in the 3 mg group by oral MB-treatment (stage 2: ctrl: 115.8±1.71 days, 3 mg: 122.2±2.06 days, 10 mg: 116.6±3.13 days, 30 mg: 113±2.91 days, 100 mg: 116.5±1.92 days; stage 3: ctrl: 123.7±1.4 days, 3 mg: 128.7±2.93 days, 10 mg: 122.4±3.85 days, 30 mg: 121.7±3.39 days, 100 mg: 126.3±2.05 days) (Fig. 3A). Accordingly, survival time was not significantly altered by oral MB-treatment, even though the death was slightly delayed by the treatment with 3 mg MB and to a lesser extent with 100 mg MB (ctrl: 129±1.15 days; 3 mg: 134±3.37 days; 10 mg: 129±4.12 days; 30 mg: 129±3.69 days; 100 mg: 132±2.41 days). Figure 3C shows the corresponding survival curves.

**Figure 4:**
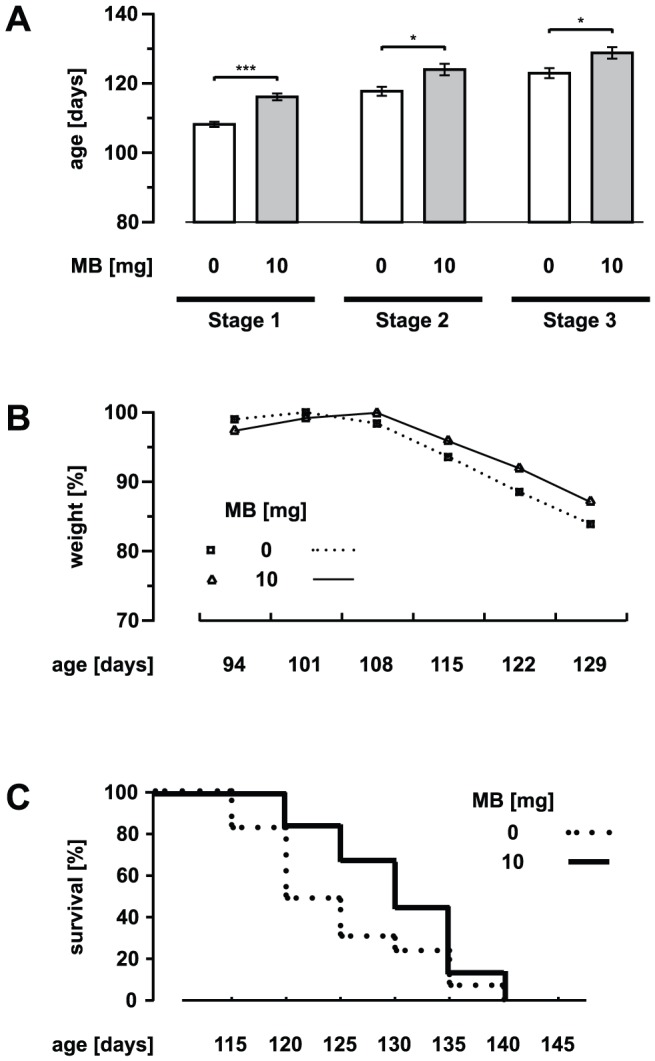
Delayed onset of disease by intraperitoneal application of MB. (A) Control and MB-treated (10 mg intraperitoneal injection per kg body weight per day) SOD1^G93A^ mice with respect to the different stages of disease. (B and C) Weight and survival profiles for SOD1^G93A^ mice (control and MB-treated). In B the data are presented relative to the highest value in each group. Values are presented as mean ± SEM; both groups: n = 16 mice; ANOVA followed by Tukey test (*p<0.05, ***p<0.001).

**Figure 5:**
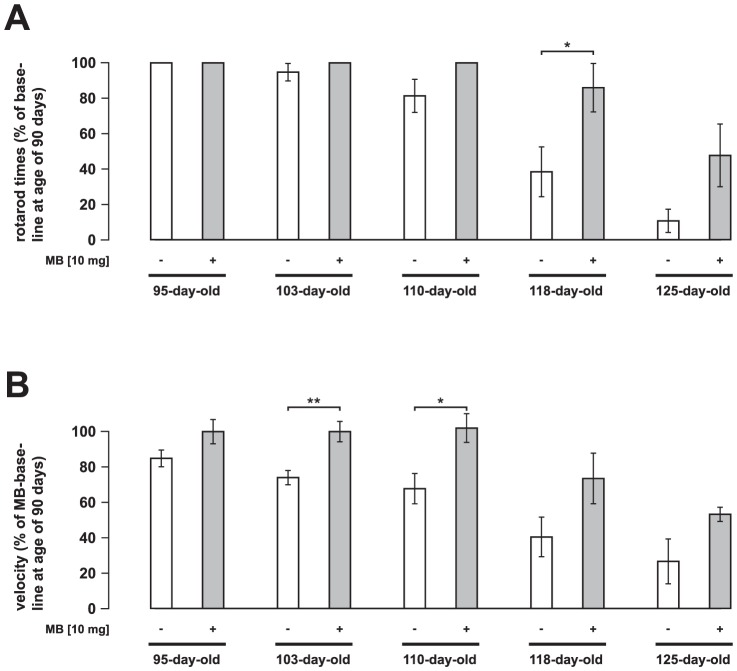
Delayed motor disturbance by MB application. Rotarod motor performance of untreated and MB-treated (10 mg intraperitoneal injection per kg body weight per day) SOD1^G93A^ mice. The time until mice fell off the rotarod at 12 rpm (A) and the velocity the mice reached using an acceleration rate of 1 rpm every 10 s (B) are presented. Each animal was tested three times per trial. Values are presented as mean ± SEM; both groups: n = 10 mice; ANOVA followed by Tukey test (*p<0.05, **p<0.01).

In another set of experiments, MB was administered by intraperitoneal injection (Fig. 4). For this purpose, an intraperitoneal dose of 10 mg MB per kg body weight per day in treatment trials of mice was chosen based on previous studies [42], [43]. Treatment with 10 mg MB delayed the onset of disease by nearly 9 days (ctrl: 107.9±0.89 days, MB: 116.4±1.16 days, p<0.001) (Fig. 4A) as was the Onset of weight loss (Fig. 4B). However, overall weight loss was not significantly changed in MB-treated mice. The start of the later disease stages was delayed by around 7 days (stage 2: ctrl: 117.4±1.45 days, MB: 124.3±1.73 days, p<0.05; stage 3: ctrl: 122.5±1.6 days, MB: 129.1±1.69 days, p<0.05) (Fig. 4A). Survival time was also extended by nearly 7 days (death: ctrl: 125.7±1.85 days, MB: 132.2±1.9 days, p<0.05). Figure 4C shows the corresponding survival curves. Correspondingly, MB treatment also delayed motor dysfunction as evaluated by rotarod performance (Fig. 5). Grip strength of the forelimbs was improved (not statistically significant) in clinical stages by the i. p. treatment (data not shown).

### Effect of systemic application of MB on microglial activation and motor neuron survival

**Figure 6:**
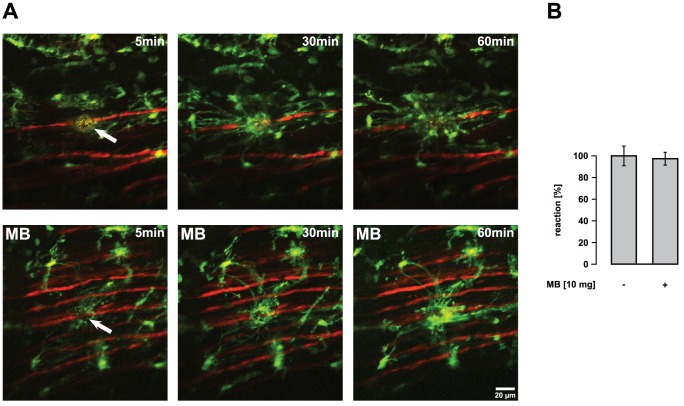
Effect of systemic application of MB on microglial reaction towards laser-induced axonal injury. Microglial reaction towards laser-induced axonal transections within the lateral column of the spinal cord was recorded. Images are arranged such that rostral is to the left side. (A) Left images were taken immediately (5 min) after axonal transection (autofluorescence and arrow) in mutant (SOD1^G93A^) mice. Respective images (middle and right) were taken 30 and 60 min after injury, respectively. Upper images represent *in vivo* recordings of an exemplary non-treated SOD1^G93A^ mouse. In the lower experiment, the mouse was treated with MB (10 mg oral per kg body weight per day; drug administration started at the age of 45 days). (B) Quantification of microglial response to the injured site. No significant differences in microglial reaction towards a laser-induced axonal injury were observed between non-treated and MB-treated SOD1^G93A^ mice. Mutant mice were 60 to 90 days of age. Values are presented as mean ± SEM; no treatment: n = 9 mice, MB-treatment: n = 8.

**Figure 7:**
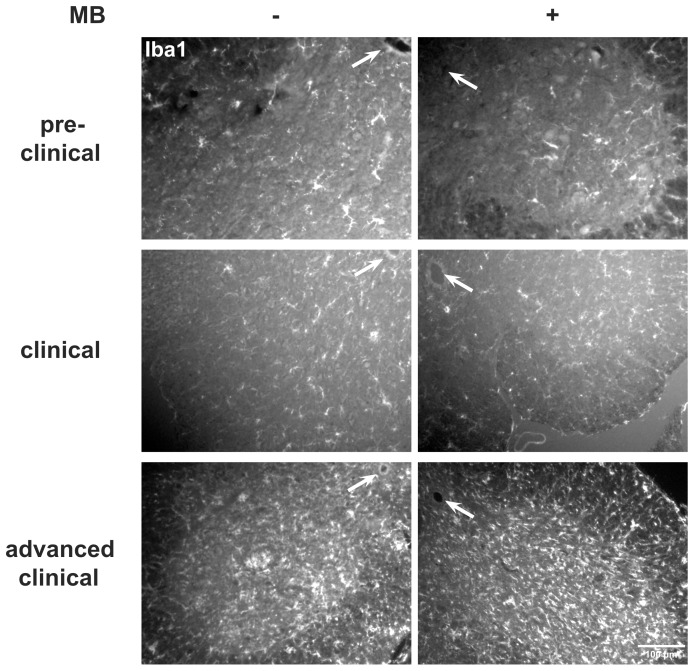
Effect of systemic application of MB on microgliosis. Iba1 staining of the anterior horn at level L3-L5 during the disease course of SOD1^G93A^. MB-treatment did not inhibit the observed microgliosis. Arrows point to the central canal.

To test the influence of systemically applied MB on microglial reaction to laser-induced axon transection within the lateral column and on motor neuron survival, we treated SOD1^G93A^ mice with MB (10 mg oral per kg body weight per day) and performed 2P-LSM recordings for microglia activation and immunohistochemistry for quantification of lumbar anterior horn neurons. No significant differences were observed in control versus treated mice when microglial reactions towards single axon transections were studied (60 to 90-day-old; control response 100±9.3%, MB-modified response 97.4±6.2%) (Fig. 6). Similarly, microglial reactions were comparable in mice at different clinical stages (data not shown). Accordingly, Iba1 immunostaining of the lumbar anterior horn showed no differences between MB-treated SOD1^G93A^ mice and controls with respect to microgliosis (Fig. 7).

**Figure 8:**
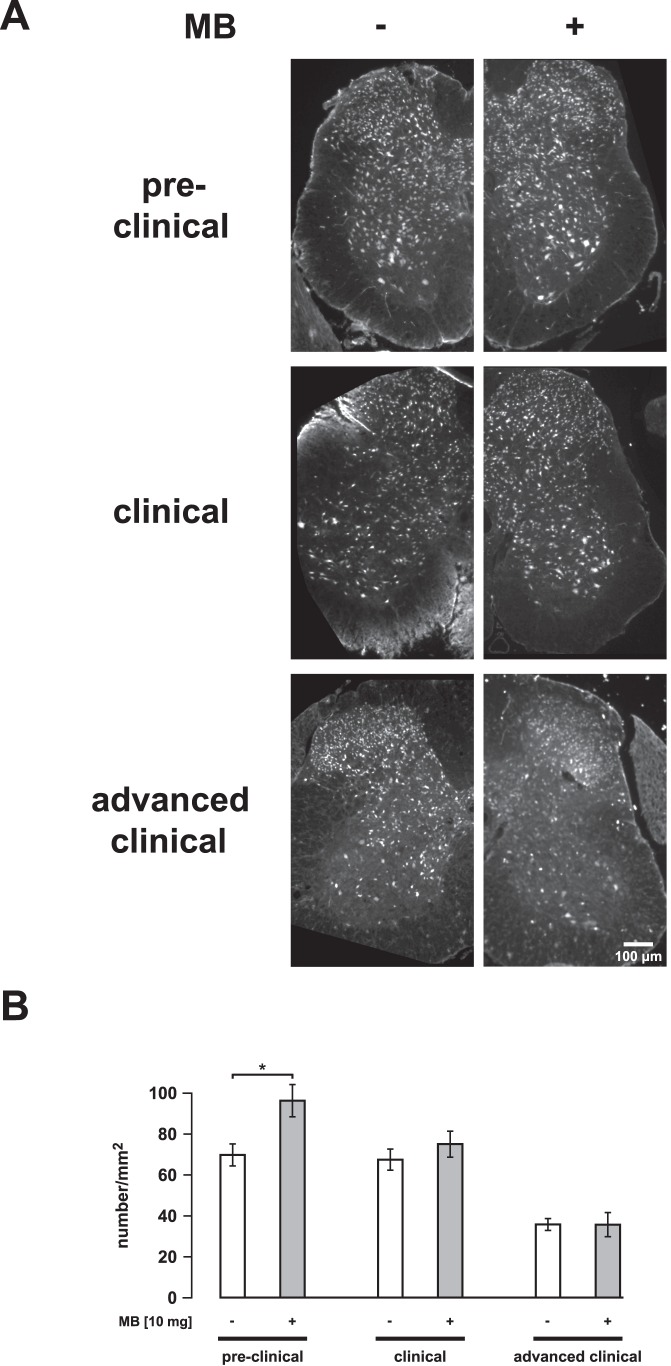
Effect of systemic application of MB on motor neuron survival. (A) NeuN-stained cross-sections of the spinal cord at the level L3 to L5. Non-treated controls (SOD1^G93A^ mice) are depicted left. Corresponding sections of MB-treated SOD1^G93A^ mice are shown on the right (10 mg oral per kg body weight per day; drug administration started at the age of 45 days) (B) Counting of neurons in the anterior horn of SOD1^G93A^ mice at different disease stages. Cell somata bigger than 20 µm were counted and given per mm^2^. In preclinical stages, neuron number was significantly higher in MB-treated mice compared to non-treated mice indicating an early neuroprotective effect of MB. Note that no differences were observed in later disease stages. Values are presented as mean ± SEM; n = 4 mice for each group; ANOVA followed by Tukey test (*p<0.05).

**Figure 9:**
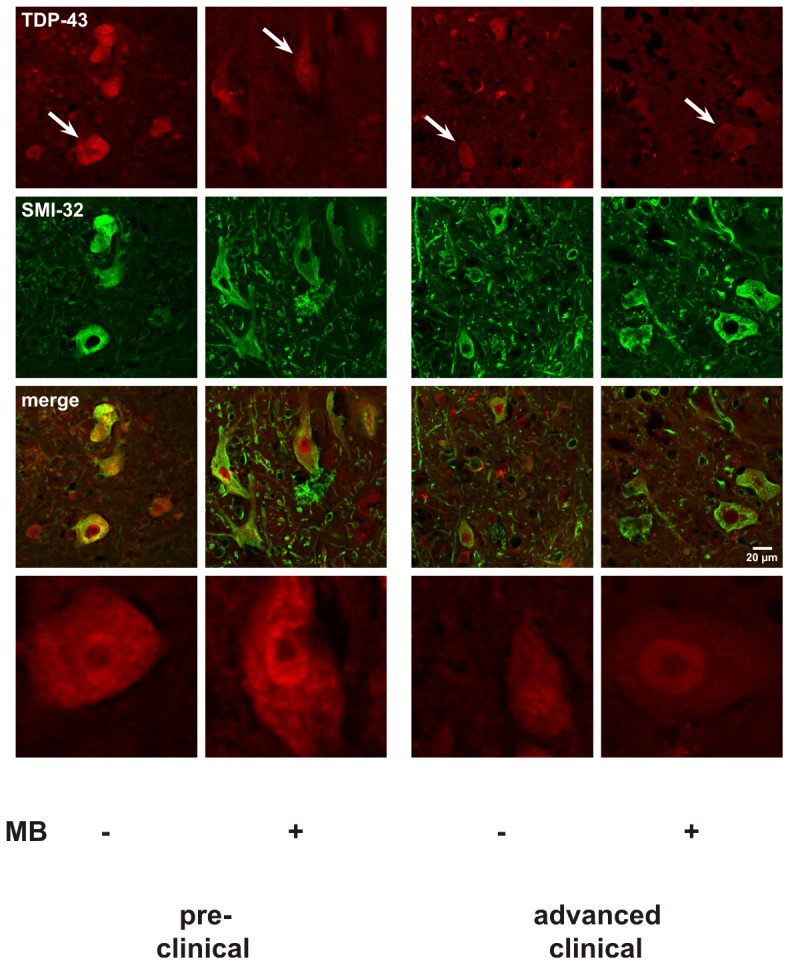
Effect of systemic application of MB on intracellular TDP-43-containing aggregates. To analyze intracellular aggregations in anterior horn neurons, TDP-43 and SMI-32 staining was performed in lumbar cross-sections. TDP-43-containing aggregates in SMI-32-positive neurons were observed in the anterior horn of control and MB-treated pre-clinical and advanced clinical mice. An exemplary TDP-43-stained neuron from each group, marked by an arrow, is shown in the last line of images.

To determine whether MB-induced increases in motor neuron survival could underlie the observed delay of disease onset, NeuN-stained cross-sections of the spinal cord at level L3 to L5 were investigated. Pre-clinically, the number of counted neurons was significantly higher in MB-treated (10 mg oral per kg body weight per day) mice than in control siblings (pre-clinical 90-day-old mice: ctrl: 70.4±5.77 per mm^2^, MB: 96.9±8.18) (Fig. 8A and B). However, this difference in neuron count was not present at the clinical and advanced clinical stages indicating only an initial and temporary effect of MB (clinical stage at 110-day-old mice: ctrl: 68.2±5.59 per mm^2^, MB: 75.8±6.71; advanced clinical stage at 130-day-old mice: ctrl: 36.5±3.2, MB: 36.4±6.25) (Fig. 8A and B). To analyze intracellular aggregations in affected motor neurons, TDP-43 and SOD1 staining was performed in lumbar cross-sections. Figure 9 shows TDP-43-containing aggregates in SMI-32-positive neurons in the anterior horn of control and MB-treated pre-clinical and advanced clinical mice. We observed no reductions of intracellular TDP-43-containing aggregates in MB-treated mice when compared with control. In pre-clinical mice, the fraction of the intracellular area filled with aggregates was 99.4±2.6% of the control value set as 100±8.1% (4 mice in each group). In clinical mice, the fraction was 101.9±7.5% of the corresponding control value set as 100±9.1% (3 mice in each group). Additionally, SOD1 staining revealed no clear reductions of intracellular SOD1-containing aggregates in lumbar anterior horn neurons (data not shown).

## Discussion

Neuroinflammation is a prominent feature of murine ALS animal models [1], [5], [9], [10], [11], [12], [44]. Despite strong evidence for microglial activation and release of pro-inflammatory cytokines driving disease progression, anti-inflammatory strategies have been overall disappointing.

Our earlier findings using *in vivo* time-lapse imaging of the spinal cord ±dorsal tracts indicated that the NOS and sGC inhibitor methylene blue (MB) strongly reduce microglial activation by inhibiting NO-mediated reactions towards tissue injury [16]. The present study confirmed this for the lateral columns of the spinal cord in both control and mutant mice. This region contains efferent (motor) fibers that undergo degeneration in ALS [45], [46]. Furthermore, addition to this local anti-inflammatory effect *in vivo*, MB at high concentration significantly inhibited the induction of cytokines and chemokines in control and mutant microglia *in vitro*. These results prompted us to study potential therapeutic effects of MB in an ALS mouse model.

MB influenced the course of the disease in SOD1^G93A^ mice when administered from preclinical stages onwards via oral or intraperitoneal routes. For both MB significantly delayed the onset of disease by nearly 10 days when, in the case of oral application, it was administered at 3 or 10 mg per kg body weight per day. The Time from onset of disease to death was not changed in either group indicating that overall disease progression was not affected. In confirmation, a recent study in which 1 or 10 mg MB per kg body weight per day was administered intraperitoneally beginning at the onset of SOD1^G93A^ and TDP-43^G348C^ disease showed no extension of the lifespan [47]. Thus, neuroprotection by MB seems to be more crucial in preclinical than in clinical stages.

A further increase of MB dose in our trial had no effect on disease course which was unexpected in view of the previously observed local anti-inflammatory effects after application of MB at high doses *in vivo* and *in vitro*. Administration at 30 or 100 mg per kg body weight per day from preclinical stages onwards had no significant effects on onset or survival, as has been recently shown by a treatment trial in which 25 mg per kg body weight per day was administered orally beginning at a similar preclinical stage [48]. Apparently, the high concentrations needed inside and locally within the central nervous tissue for anti-inflammatory effects cannot be achieved by increasing the dose of systematically administered MB. Moreover, increasing the dose actually led to a loss of the beneficial neuroprotective effects of MB when administered at 3 or 10 mg per kg body weight per day. The reason for this neutralization is unclear. Similar loss of beneficial effects has been shown, for instance, for imipramine in mdx mice [49]. As known from human clinical trials, the dose of a drug has to be optimized as an increase may abolish beneficial effects, due, for instance, by inducing unwanted side effects.

Earlier studies indicated that microglial activation in ALS models influences disease progression rather than delaying the onset of disease [1], [9], [10]. Accordingly, in our *in vivo* studies using *in vivo* time-lapse imaging, we observed that microglial responses were not affected in MB-treated mice. In particular, the microglial reaction towards local laser lesions was unaltered, compared to non-treated SOD1^G93A^ mice, indicating that MB-treatment did not affect microglial activity, and in turn microgliosis was not changed by MB treatment. Our data therefore suggest that the effect of MB may not be a direct consequence of microglial inhibition but rather, an affect on neuronal survival. The observed rescue of motor neurons in pre-clinical stages also supports a neuroprotective effect of MB.

The observed protective effect seems not only to be primarily microglia-independent but also independent of the signal molecule NO, since application of a broad-spectrum NOS inhibitor to SOD1^G93A^ mice had no effect on the course of disease [50]. Several cellular and molecular mechanisms have been attributed to the mode of action of MB. Although modulation of the NO pathway appears to be its main effect, various other cellular and molecular targets have been described [20], [40], [47]. Most interestingly, MB can inhibit amyloid Aβ oligomerization and thus reduce Aβ oligomer formation, a major hallmark of AD [51]. This led to a phase II trial using MB for AD patients that showed positive effects on memory function [21]. In another study, MB inhibited the aggregation of TAR DNA-binding protein 43 (TDP-43) *in vitro* [52], [53]. This finding indicates that MB may also have neuroprotective effects by reducing aggregate formation or by dissolving existing aggregates in neurodegenerative diseases. In ALS, tau-negative and ubiquitine-positive inclusions are observed, with TDP-43 being a major aggregate component [54], [55]. Even though our study supports the hypothesis of a neuroprotective effect of MB, immunohistochemistrical investigations showed no clear alterations of TDP-43-containing aggregates in lumbar anterior horn neurons by MB treatment. In the recently published study of Audet et al. [47], SOD1 and TDP-43 aggregations were nearly unchanged in the respective mouse models for ALS after treatment with MB. Further studies are therefore necessary to unravel the neuroprotective effect of MB in ALS mice.

Summing up, potentially beneficial effects of locally applied MB on inflammatory events contributing to disease progression could not be reproduced in SOD1^G93A^ mice via systemic administration, whereas systemic MB application delayed disease onset via neuroprotection. *In vivo* imaging turns out to be an appropriate tool to establish if a supposed anti-inflammatory action of a drug really exists after systemic application. MB is known to penetrate the blood-brain-barrier [56], [57] and seems to exert neuroprotective effects in an ALS mouse model as in the case of AD. MB may therefore represent an already well-studied drug for further investigation in ALS animal models, and potentially safe for human trials.

## References

[1] Boillee S, Yamanaka K, Lobsiger CS, Copeland NG, Jenkins NA, et al (2006) Onset and Progression in Inherited ALS Determined by Motor Neurons and Microglia. Science 312: 1389–1392. 16741123 10.1126/science.1123511

[2] Rosen DR, Siddique T, Patterson D, Figlewicz DA, Sapp P, et al (1993) Mutations in Cu/Zn superoxide dismutase gene are associated with familial amyotrophic lateral sclerosis. Nature 362: 59–62. 8446170 10.1038/362059a0

[3] Gurney ME, Pu H, Chiu AY, Dal Canto MC, Polchow CY, et al (1994) Motor neuron degeneration in mice that express a human Cu,Zn superoxide dismutase mutation. Science 264: 1772–1775. 8209258 10.1126/science.8209258

[4] Wong PC, Borchelt DR (1995) Motor neuron disease caused by mutations in superoxide dismutase 1. Curr Opin Neurol 8: 294–301. 7582045 10.1097/00019052-199508000-00008

[5] Boillee S, Vande Velde C, Cleveland DW (2006) ALS: a disease of motor neurons and their nonneuronal neighbors. Neuron 52: 39–59. 17015226 10.1016/j.neuron.2006.09.018

[6] Neusch C, Bahr M, Schneider-Gold C (2007) Glia cells in amyotrophic lateral sclerosis: new clues to understanding an old disease? Muscle Nerve 35: 712–724. 17373702 10.1002/mus.20768

[7] Chattopadhyay M, Valentine JS (2009) Aggregation of copper-zinc superoxide dismutase in familial and sporadic ALS. Antioxid Redox Signal 11: 1603–1614. 19271992 10.1089/ars.2009.2536 PMC2842589

[8] Synofzik M, Fernandez-Santiago R, Maetzler W, Schols L, Andersen PM (2010) The human G93A SOD1 phenotype closely resembles sporadic amyotrophic lateral sclerosis. J Neurol Neurosurg Psychiatry 81: 764–767. 20176600 10.1136/jnnp.2009.181719

[9] Beers DR, Henkel JS, Xiao Q, Zhao W, Wang J, et al (2006) Wild-type microglia extend survival in PU.1 knockout mice with familial amyotrophic lateral sclerosis. Proc Natl Acad Sci U S A 103: 16021–16026. 17043238 10.1073/pnas.0607423103 PMC1613228

[10] Lobsiger CS, Cleveland DW (2007) Glial cells as intrinsic components of non-cell-autonomous neurodegenerative disease. Nat Neurosci 10: 1355–1360. 17965655 10.1038/nn1988 PMC3110080

[11] Yamanaka K, Chun SJ, Boillee S, Fujimori-Tonou N, Yamashita H, et al (2008) Astrocytes as determinants of disease progression in inherited amyotrophic lateral sclerosis. Nat Neurosci 11: 251–253. 18246065 10.1038/nn2047 PMC3137510

[12] Yamanaka K, Boillee S, Roberts EA, Garcia ML, McAlonis-Downes M, et al (2008) Mutant SOD1 in cell types other than motor neurons and oligodendrocytes accelerates onset of disease in ALS mice. Proc Natl Acad Sci U S A 105: 7594–7599. 18492803 10.1073/pnas.0802556105 PMC2396671

[13] Nimmerjahn A, Kirchhoff F, Helmchen F (2005) Resting microglial cells are highly dynamic surveillants of brain parenchyma in vivo. Science 308: 1314–1318. 15831717 10.1126/science.1110647

[14] Davalos D, Grutzendler J, Yang G, Kim JV, Zuo Y, et al (2005) ATP mediates rapid microglial response to local brain injury in vivo. Nat Neurosci 8: 752–758. 15895084 10.1038/nn1472

[15] Hanisch UK, Kettenmann H (2007) Microglia: active sensor and versatile effector cells in the normal and pathologic brain. Nat Neurosci 10: 1387–1394. 17965659 10.1038/nn1997

[16] Dibaj P, Nadrigny F, Steffens H, Scheller A, Hirrlinger J, et al (2010) NO mediates microglial response to acute spinal cord injury under ATP control in vivo. Glia 58: 1133–1144. 20468054 10.1002/glia.20993

[17] Dibaj P, Steffens H, Nadrigny F, Neusch C, Kirchhoff F, et al (2010) Long-lasting post-mortem activity of spinal microglia in situ in mice. J Neurosci Res 88: 2431–2440. 20623536 10.1002/jnr.22402

[18] Duan Y, Sahley CL, Muller KJ (2009) ATP and NO dually control migration of microglia to nerve lesions. Dev Neurobiol 69: 60–72. 19025930 10.1002/dneu.20689 PMC2748121

[19] Dibaj P, Steffens H, Zschuntzsch J, Nadrigny F, Schomburg ED, et al (2011) In Vivo imaging reveals distinct inflammatory activity of CNS microglia versus PNS macrophages in a mouse model for ALS. PLoS One 6: e17910. 21437247 10.1371/journal.pone.0017910 PMC3060882

[20] Oz M, Lorke DE, Hasan M, Petroianu GA (2009) Cellular and molecular actions of methylene blue in the nervous system. Med Res Rev. 10.1002/med.20177 PMC3005530 19760660

[21] Gura T (2008) Hope in Alzheimer's fight emerges from unexpected places. Nat Med 14: 894. 18776868 10.1038/nm0908-894

[22] Oz M, Lorke DE, Petroianu GA (2009) Methylene blue and Alzheimer's disease. Biochem Pharmacol 78: 927–932. 19433072 10.1016/j.bcp.2009.04.034

[23] Guegan C, Przedborski S (2003) Programmed cell death in amyotrophic lateral sclerosis. J Clin Invest 111: 153–161. 12531867 10.1172/JCI17610 PMC151885

[24] Hegedus J, Putman CT, Gordon T (2007) Time course of preferential motor unit loss in the SOD1 G93A mouse model of amyotrophic lateral sclerosis. Neurobiol Dis 28: 154–164. 17766128 10.1016/j.nbd.2007.07.003

[25] Dibaj P, Steffens H, Zschuntzsch J, Kirchhoff F, Schomburg ED, et al (2011) In vivo imaging reveals rapid morphological reactions of astrocytes towards focal lesions in an ALS mouse model. Neurosci Lett 497: 148–151. 21539893 10.1016/j.neulet.2011.04.049

[26] Jung S, Aliberti J, Graemmel P, Sunshine MJ, Kreutzberg GW, et al (2000) Analysis of fractalkine receptor CX(3)CR1 function by targeted deletion and green fluorescent protein reporter gene insertion. Mol Cell Biol 20: 4106–4114. 10805752 10.1128/mcb.20.11.4106-4114.2000 PMC85780

[27] Hirrlinger PG, Scheller A, Braun C, Quintela-Schneider M, Fuss B, et al (2005) Expression of reef coral fluorescent proteins in the central nervous system of transgenic mice. Mol Cell Neurosci 30: 291–303. 16169246 10.1016/j.mcn.2005.08.011

[28] Hegedus J, Putman CT, Gordon T (2009) Progressive motor unit loss in the G93A mouse model of amyotrophic lateral sclerosis is unaffected by gender. Muscle Nerve 39: 318–327. 19208415 10.1002/mus.21160

[29] Kaiser M, Maletzki I, Hulsmann S, Holtmann B, Schulz-Schaeffer W, et al (2006) Progressive loss of a glial potassium channel (KCNJ10) in the spinal cord of the SOD1 (G93A) transgenic mouse model of amyotrophic lateral sclerosis. J Neurochem 99: 900–912. 16925593 10.1111/j.1471-4159.2006.04131.x

[30] De Luca A, Pierno S, Liantonio A, Cetrone M, Camerino C, et al (2003) Enhanced dystrophic progression in mdx mice by exercise and beneficial effects of taurine and insulin-like growth factor-1. J Pharmacol Exp Ther 304: 453–463. 12490622 10.1124/jpet.102.041343

[31] Golumbek PT, Keeling RM, Connolly AM (2007) Strength and corticosteroid responsiveness of mdx mice is unchanged by RAG2 gene knockout. Neuromuscul Disord 17: 376–384. 17452104 10.1016/j.nmd.2007.02.005

[32] Liebetanz D, Hagemann K, von Lewinski F, Kahler E, Paulus W (2004) Extensive exercise is not harmful in amyotrophic lateral sclerosis. Eur J Neurosci 20: 3115–3120. 15579165 10.1111/j.1460-9568.2004.03769.x

[33] Carreras I, Yuruker S, Aytan N, Hossain L, Choi JK, et al (2011) Moderate exercise delays the motor performance decline in a transgenic model of ALS. Brain Res 1313: 192–201. 10.1016/j.brainres.2009.11.051 PMC2892864 19968977

[34] Saywell SA, Ford TW, Meehan CF, Todd AJ, Kirkwood PA (2010) Electrophysiological and morphological characterization of propriospinal interneurons in the thoracic spinal cord. J Neurophysiol 105: 806–826. 21106900 10.1152/jn.00738.2010 PMC3059177

[35] Unal-Cevik I, Kilinc M, Gursoy-Ozdemir Y, Gurer G, Dalkara T (2004) Loss of NeuN immunoreactivity after cerebral ischemia does not indicate neuronal cell loss: a cautionary note. Brain Res 1015: 169–174. 15223381 10.1016/j.brainres.2004.04.032

[36] McPhail LT, McBride CB, McGraw J, Steeves JD, Tetzlaff W (2004) Axotomy abolishes NeuN expression in facial but not rubrospinal neurons. Exp Neurol 185: 182–190. 14697329 10.1016/j.expneurol.2003.10.001

[37] van Rossum D, Hilbert S, Strassenburg S, Hanisch UK, Bruck W (2008) Myelin-phagocytosing macrophages in isolated sciatic and optic nerves reveal a unique reactive phenotype. Glia 56: 271–283. 18069669 10.1002/glia.20611

[38] Regen T, van Rossum D, Scheffel J, Kastriti ME, Revelo NH, et al (2011) CD14 and TRIF govern distinct responsiveness and responses in mouse microglial TLR4 challenges by structural variants of LPS. Brain Behav Immun 25: 957–970. 20951794 10.1016/j.bbi.2010.10.009

[39] Dibaj P, Steffens H, Nadrigny F, Kirchhoff F, Schomburg ED (2011) Purinergic activation of dorsal root ganglion neurones in vivo. Neurosci Lett 487: 107–109. 20937358 10.1016/j.neulet.2010.10.005

[40] Duan Y, Haugabook SJ, Sahley CL, Muller KJ (2003) Methylene blue blocks cGMP production and disrupts directed migration of microglia to nerve lesions in the leech CNS. J Neurobiol 57: 183–192. 14556284 10.1002/neu.10262

[41] Walter-Sack I, Rengelshausen J, Oberwittler H, Burhenne J, Mueller O, et al (2009) High absolute bioavailability of methylene blue given as an aqueous oral formulation. Eur J Clin Pharmacol 65: 179–189. 18810398 10.1007/s00228-008-0563-x

[42] Barbe C, Rochetaing A, Kreher P (2002) Mechanisms underlying the coronary vasodilation in the isolated perfused hearts of rats submitted to one week of high carbon monoxide exposure in vivo. Inhal Toxicol 14: 273–285. 12028817 10.1080/08958370252809059

[43] Kulkarni SK, Dhir A (2007) Possible involvement of L-arginine-nitric oxide (NO)-cyclic guanosine monophosphate (cGMP) signaling pathway in the antidepressant activity of berberine chloride. Eur J Pharmacol 569: 77–83. 17585901 10.1016/j.ejphar.2007.05.002

[44] Boillee S, Lobsiger CS (2008) [Glial cells not that supportive for motor neurons]. Med Sci (Paris) 24: 124–126. 18272066 10.1051/medsci/2008242124

[45] Yamanaka K, Miller TM, McAlonis-Downes M, Chun SJ, Cleveland DW (2006) Progressive spinal axonal degeneration and slowness in ALS2-deficient mice. Ann Neurol 60: 95–104. 16802286 10.1002/ana.20888

[46] Ozdinler PH, Benn S, Yamamoto TH, Guzel M, Brown RH Jr, et al (2011) Corticospinal motor neurons and related subcerebral projection neurons undergo early and specific neurodegeneration in hSOD1G(3)A transgenic ALS mice. J Neurosci 31: 4166–4177. 21411657 10.1523/JNEUROSCI.4184-10.2011 PMC3643523

[47] Audet JN, Soucy G, Julien JP (2012) Methylene blue administration fails to confer neuroprotection in two amyotrophic lateral sclerosis mouse models. Neuroscience 209: 136–143. 22230045 10.1016/j.neuroscience.2011.12.047

[48] Lougheed R, Turnbull J (2011) Lack of effect of methylene blue in the SOD1 G93A mouse model of amyotrophic lateral sclerosis. PLoS One 6: e23141. 21998625 10.1371/journal.pone.0023141 PMC3188547

[49] Carre-Pierrat M, Lafoux A, Tanniou G, Chambonnier L, Divet A, et al (2011) Pre-clinical study of 21 approved drugs in the mdx mouse. Neuromuscul Disord 21: 313–327. 21392993 10.1016/j.nmd.2011.01.005

[50] Martinez JA, Francis GJ, Liu WQ, Pradzinsky N, Fine J, et al (2008) Intranasal delivery of insulin and a nitric oxide synthase inhibitor in an experimental model of amyotrophic lateral sclerosis. Neuroscience 157: 908–925. 18951954 10.1016/j.neuroscience.2008.08.073

[51] Necula M, Kayed R, Milton S, Glabe CG (2007) Small molecule inhibitors of aggregation indicate that amyloid beta oligomerization and fibrillization pathways are independent and distinct. J Biol Chem 282: 10311–10324. 17284452 10.1074/jbc.M608207200

[52] Yamashita M, Nonaka T, Arai T, Kametani F, Buchman VL, et al (2009) Methylene blue and dimebon inhibit aggregation of TDP-43 in cellular models. FEBS Lett 583: 2419–2424. 19560462 10.1016/j.febslet.2009.06.042

[53] Arai T, Hasegawa M, Nonoka T, Kametani F, Yamashita M, et al (2010) Phosphorylated and cleaved TDP-43 in ALS, FTLD and other neurodegenerative disorders and in cellular models of TDP-43 proteinopathy. Neuropathology 30: 170–181. 20102522 10.1111/j.1440-1789.2009.01089.x

[54] Neumann M, Sampathu DM, Kwong LK, Truax AC, Micsenyi MC, et al (2006) Ubiquitinated TDP-43 in frontotemporal lobar degeneration and amyotrophic lateral sclerosis. Science 314: 130–133. 17023659 10.1126/science.1134108

[55] Braak H, Ludolph A, Thal DR, Del Tredici K (2010) Amyotrophic lateral sclerosis: dash-like accumulation of phosphorylated TDP-43 in somatodendritic and axonal compartments of somatomotor neurons of the lower brainstem and spinal cord. Acta Neuropathol 120: 67–74. 20379728 10.1007/s00401-010-0683-0

[56] O'Leary JL, Petty J, Harris AB, Inukai J (1968) Supravital staining of mammalian brain with intra-arterial methylene blue followed by pressurized oxygen. Stain Technol 43: 197–201. 4175125 10.3109/10520296809115068

[57] Peter C, Hongwan D, Kupfer A, Lauterburg BH (2000) Pharmacokinetics and organ distribution of intravenous and oral methylene blue. Eur J Clin Pharmacol 56: 247–250. 10952480 10.1007/s002280000124

